# Dynamic-Cross-Linked, Regulated, and Controllable Mineralization Degree and Morphology of Collagen Biomineralization

**DOI:** 10.3390/jfb15120356

**Published:** 2024-11-22

**Authors:** Ziyao Geng, Fan Xu, Ying Liu, Aike Qiao, Tianming Du

**Affiliations:** Beijing International Science and Technology Cooperation Base for Intelligent Physiological Measurement and Clinical Transformation, Department of Biomedical Engineering, College of Chemistry and Life Sciences, Beijing University of Technology, Beijing 100124, China; gengziyao@emails.bjut.edu.cn (Z.G.); 15601352718@163.com (F.X.); 13120284885@163.com (Y.L.); qak@bjut.edu.cn (A.Q.)

**Keywords:** collagen, biomineralization, dynamic cross-linking, mechanical property, hydroxyapatite crystal

## Abstract

The cross-linking process of collagen is one of the more important ways to improve the mineralization ability of collagen. However, the regulatory effect of dynamic cross-linking on biomineralization in vitro remains unclear. Dynamic-cross-linked mineralized collagen under different cross-linking processes, according to the process of cross-linking and mineralization of natural bone, was prepared in this study. Mineralization was performed for 12 h at 4, 8, and 12 h of collagen cross-linking. Scanning electron microscopy (SEM) and transmission electron microscopy (TEM) showed the characteristics of dynamic-cross-linked mineralization in terms of morphological transformation and distribution. Fourier transform infrared spectroscopy (FTIR) analysis showed the crystallinity characteristics of the hydroxyapatite (HA) crystal formation. Pre-cross-linked dynamic-cross-linked mineralization refers to the process of cross-linking for a period of time and then side cross-linked mineralization. The mineral content, enzyme stability, and mechanical properties of mineralized collagen were improved through a dynamic cross-linking process of pre-cross-linking. The swelling performance was reduced through the dynamic cross-linking process of pre-cross-linking. This study suggests that the dynamic cross-linking process through pre-cross-linking could make it easier for minerals to permeate and deposit between collagen fibers, improve mineralization efficiency, and, thus, enhance the mechanical strength of biomineralization. This study can provide new ideas and a theoretical basis for designing mineralized collagen scaffolds with better bone repair ability.

## 1. Introduction

Bone tissue is a composite structure composed of inorganic matter and organic matter, and it has good stiffness and toughness. Bone is one of the most complex instances of biomineralization in nature. In the process of natural bone formation, osteoblasts first synthesize collagen polypeptide spiral chains, and they then self-assemble collagen fibril by the amino acid hydroxylation modification of peptide chains [1]. Collagen fibers are then released into the interstitial fluid, where mineralization processes occur with calcium and phosphate ions in the interstitial fluid [2]. In this process, the formation and mineralization of collagen fibers are continuously carried out. At the same time, after the formation of collagen fibers, collagen fibers are successively assembled into collagen fiber bundles through multiple stages and are cross-linked to form fiber network structures [3]. Therefore, cross-linking and mineralization simultaneously occur in natural bone, that is, the dynamic cross-linking process. Eventually, in a dynamic process, mineralized collagen further assembles to form high-strength, multi-scale bone. At present, the influence of the dynamic cross-linking process on mineralization is rarely discussed.

Currently, one of the most effective methods for treating bone defects is to implant mineralized collagen bone material scaffolds [4,5]. In biomineralization in vitro, the phosphorylation degree, cross-linking, diameter, surface charge, and other properties of collagen directly affect the mineralization process [6,7]. Among them, cross-linking has been widely proven to be one of the most effective ways to improve the mechanical strength of materials. Li et al. used a polymer-induced liquid precursor mineralization process to prepare collagen–hydroxyapatite composites, and they demonstrated that cross-linking actually enhances collagen mineralization [8]. The experiments of Du et al. [9] proved that increasing the cross-linking degree of collagen could provide better mechanical and structural conditions for HA deposition, ultimately improving the mineralization ability of collagen. The improvement of mineralization is also the key for improving the mechanical properties of mineralized collagen. It can be seen that cross-linking is essential in the formation of mineralized collagen in vitro. At present, the mechanical strength of mineralized collagen materials is insufficient. Therefore, it has become the focus of current research due to the difficulty in rationally regulating biomineralization in vitro so as to form mineralized collagen that is consistent with the mineralized structure and mechanical strength of natural bone in vitro [10].

Currently, there are numerous commonly used cross-linking methods. For example, dehydrogenated hydrothermal solution (DHT) is a simple method for cross-linking collagen molecules, which requires exposing collagen molecules to a high temperature (>90 °C) under vacuum conditions [11]. However, the cross-linking reaction time induced by DHT is too long. UV-induced cross-linking is faster and more efficient than DHT treatment [12]. However, ultraviolet light alone cannot produce high cross-linking densities. In experiments, EDC/NHS (N-hydroxysuccinimide) is often used as a cross-linking agent to cross-link collagen. However, the current cross-linking methods are static. These cross-linking methods are still not able to prepare high-strength biomineralized scaffolds similar to those found in nature.

**Figure 1:**
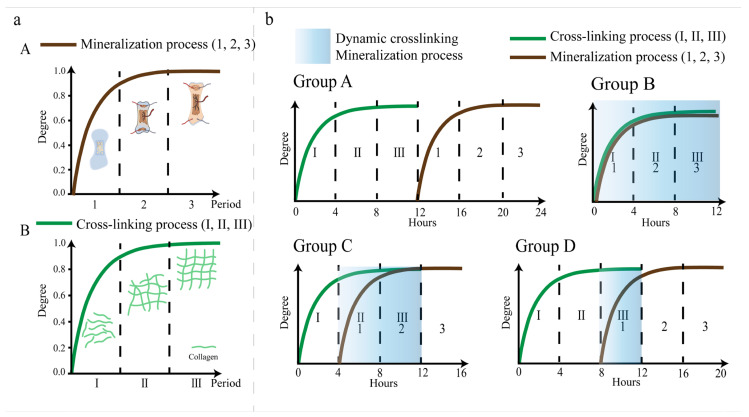
A schematic diagram of the collagen cross-linking and mineralization processes. (**a**) The mineralization process of natural bone is non-linear and can be divided into three stages (A) [13]. The cross-linking process of natural bone is also a nonlinear process in three stages (B). (**b**) Four sets of experiments were set up to explore the effect of dynamic cross-linking on mineralization. Group A was fully cross-linked for 12 h and mineralized for 12 h. Group B involved cross-linked mineralization that was simultaneously carried out for 12 h. Group C involved cross-linking for 4 h and then cross-linked mineralization for 12 h. Group D involved cross-linking for 8 h and then cross-linked mineralization for 12 h.

Based on this, it is essential to incorporate dynamic cross-linking into in vitro biomineralization. The mineralization and cross-linking processes in natural bone are divided into three stages [13,14,15] (Figure 1a). In this study, according to the natural bone mineralization process and cross-linking process, the time spent in adding mineralized fluid during dynamic cross-linking was determined in order to achieve the effect of dynamic cross-linking. Specifically, collagen was cross-linked using EDC/NHS. Then, the mineralized fluid was added according to the different processes of cross-linking so as to prepare the dynamic cross-linking mineralized collagen material. It is made to mimic the process of natural bone as much as possible, thus promoting the preparation of mineralized collagen scaffolds for bone repair.

The morphology and distribution of minerals at different scales were analyzed by scanning electron microscopy (SEM) and transmission electron microscopy (TEM). The distribution of mineral elements in the sample was analyzed by energy dispersive X-ray spectroscopy (EDS). The crystallinity and mineral content of the mineralized collagen were analyzed by Fourier transform infrared spectroscopy (FTIR) and thermogravimetry (TG). In addition, a mechanical test was used to evaluate the mechanical properties of the mineralized collagen materials, an enzymatic hydrolysis test was used to evaluate the enzymatic hydrolysis properties of the mineralized collagen materials, and a swelling test was used to evaluate the swelling rate of the mineralized collagen materials. The objective was to reveal the dynamic matching mechanism between the cross-linking process and the mineralization process of the natural bone matrix, as well as to analyze the effect of the dynamic cross-linking mineralization mechanism on the properties of bone repair materials. Dynamic cross-linking was used to promote the mineralization efficiency, thereby improving the mechanical strength of the biomineralization to achieve better bone repair results. This study could provide new ideas for designing a mineralized collagen scaffold with better mechanical strength.

## 2. Materials and Methods

### 2.1. Preparation of Cross-Linked and Mineralized Collagen

The mineralization process of natural bone is non-linear and can be divided into three stages (Figure 1a). After a period of delay, mineralization first develops rapidly, followed by continuous slow mineral formation, and it finally then reaches complete mineralization [13]. In this process, due to the influence of the formation rate of HA and the removal efficiency of nucleators, the degree of mineralization does not increase linearly with time, reflecting the non-linear dynamics of mineralization. It starts from the lagging stage where there is a bone-like but no obvious mineralization. This is followed by rapid primary mineralization, finishing with a secondary mineralization that is characterized by a sustained and slow increase in bone mineral content [13]. The cross-linking process is also non-linear (Figure 1a) [14,15]. In the initial phase of the reaction, rapid cross-linking is usually observed because of the large number of reaction sites available between the cross-linkers and collagen. At this stage, the cross-linking rate is very high. As the reaction progresses, the available reaction sites begin to decrease and the rate of cross-linking will slow down and eventually reach a saturated cross-linking state.

The collagen source solution was 1.5% bovine Achilles tendon type I collagen, and it was purchased from Chengdu Kele Biological Products, Chengdu, China. The EDC, 1-(3-Dimethylaminopropyl)-3-ethylcarbodiimide hydrochloride used was from MREDA (Beijing, China). The rest of the chemicals were obtained from Sigma-Aldrich Company (St. Louis, MI, USA).

A total of 1 mL of 32 mg/mL EDC and 1 mL of 8 mg/mL NHS was added into the 2 mL of 8 mg/mL type I collagen solution in accordance with an EDC: NHS: collagen mass ratio of 4:1:2. Next, 1 mL of 17.8 mg/mL CaCl_2_ was added to make the calcium ion concentration in the solution 0.02 M. The mixed liquor was fully stirred at the rate of 50–80 r/min at room temperature for 10 min. Then, 1 M of sodium hydroxide and 0.1 M of sodium hydroxide was used to adjust the pH to 7.4.

According to the process of collagen cross-linking and collagen mineralization [13,14,15], 1 mL of 12.6 mg/mL (NH_4_)_2_HPO_4_ was added at different times (the same time, 4 h, and 8 h) after cross-linking (Figure 1b). The pre-cross-linking time, mineralization time, and dynamic cross-linking time of each group are shown in Table (Table 1). The ratio of the amount of calcium and phosphorus in the mineralization system was 1.67. After mineralization for 12 h, the solution was centrifuged at 8000 rpm for 5 min and frozen at −50 °C by a high-speed refrigerated centrifuge (Shanghai Fulgol Analytical Instrument, Shanghai, China). After freezing for 24 h, the solution was freeze-dried at −55 °C and −85 °C in a vacuum of < 6 Pa and a freeze-drying time of 24 h, which was achieved by a vacuum freeze dryer (Beijing Biocool Instrument, Beijing, China).

### 2.2. Scanning Electron Microscopy (SEM) Observation and Energy Dispersive X-Ray Spectroscopy (EDS)

SEM (FEI Company, Quanta 650 FEG, Hilsboro, OR, USA) was used to examine the morphology of the cross-linked and mineralized collagen with different microstructures. The freeze-dried collagen samples were cut into small pieces with a side length of 5 mm and a thickness of 1 mm, and they were then fixed on the sample stage with conductive tapes. Afterward, the sample stage with the collagen specimens was sputtered and coated with a layer of gold using a vibrating polishing machine (Buehler, Lake Bluff, IL, USA) at 0.1 Torr and 15 mA for 120 s. The cross-section of the sample was observed by SEM under the condition of an accelerating voltage of 10 kV. At the same time, EDS was performed for energy spectrum analysis during SEM. The cross-section of the cross-linked and mineralized collagen scaffold was roughly a circle. The area 2 mm from the center of the circle was defined as the edge part. The edge part of the cross-section of the collagen scaffold was observed.

### 2.3. Transmission Electron Microscopy (TEM) and Selected Area Electron Diffraction (SAED)

A total of 5 mg of freeze-dried mineralized collagen samples with 1 mg of KBr were ground, crushed, and mixed with purified water in a ratio of 1:100. A total of 20 μL of the mixed suspension was dripped onto the copper mesh (ZhongMirror Science Instrument, Beijing, China) and air-dried. TEM (JEOL, JEM-2100, Akishima, Japan) was used to examine the morphology of the mineralized collagen at a voltage of 200 kV, and SAED was utilized to investigate the crystal characteristics of the mineralized collagen, including HA lattice fringes and crystalline diffraction rings of mineralized collagen.

### 2.4. Mechanical Tests

The cross-linked and mineralized collagen samples were cut into 5.6 mm-diameter and 2.5 mm-height cylinders. The samples were compressed by a universal material testing machine (INSTRON, 5944, Norwood, MA, USA) at a speed of 0.05 mm/s and a compression force of 20 N. The force–displacement curve was measured. The column chart of the elastic modulus was calculated and analyzed according to the measured results.

### 2.5. Fourier Transform Infrared Spectroscopy (FTIR) Measurements

FTIR (Bruker, Bremen, Germany) was used to investigate the chemical compositions and the crystallinity phases of the cross-linked and mineralized collagen. The FTIR measurements were performed using the potassium bromide (KBr) tablets. The ratio of collagen specimens to KBr was controlled at 1:100. All of the spectra were recorded at 4 cm^−1^ resolution intervals in the range of 400 to 4000 cm^−1^, and the spectrograms represent 32 scans.

### 2.6. Thermogravimetry (TG) Measurements

TG/DTA thermogravimetric analyzer (Seiko Instruments Inc., Chiba, Japan) was used to measure the thermal weight loss of the mineralized collagen in order to analyze the mineralization degree and structural stability. The heating temperature ranged from room temperature to 800 °C at a heating rate of 10 °C min^−1^ under the N_2_ protective gas.

### 2.7. Enzyme Measurement

The cross-linked and mineralized collagen sample were cut into 5.6 mm × 5 mm cylinders and immersed in 1M PBS solution. The mass of the sample was measured after 1 h, and it was then immersed in 0.25% trypsin solution for 24 h. In order to simulate the enzymatic hydrolysis environment in vivo, the sample was placed in a water bath at 37 °C, and the mass of the sample was measured after 24 h. The enzymatic hydrolysis rate was determined using the subsequent formula:

(1) $$Enzymolysis\;rate=\frac{m1-m0}{m0}\times 100\%$$

where *m*1 is the weight of the film after enzymatic hydrolysis, and *m*0 is the weight of the film after immersed in 1 M PBS solution.

### 2.8. Swelling Rate Measurement

The effects of the different cross-linking conditions on the swelling rate of mineralized collagen scaffold were determined by gravimetry. After freeze drying, the sample was accurately weighed and then completely immersed in 1 × PBS buffer (pH = 7.4) at room temperature (25~27 °C) until the swelling mass no longer changed. Filter paper was used to remove the surface adsorbed water and was then weighed. The swelling rate was determined using the subsequent formula:

(2) $$Swelling\;rate=\frac{Ww-Wd}{Wd}\times 100\%$$

where *Wd* is the weight before swelling, and *Ww* is the weight after swelling.

### 2.9. Statistical Methods

In order to compare the data of each group, a variance analysis of the dynamic cross-linking was performed on the mechanical test and enzyme measurement results. Statistical analysis was performed using one-way analysis of variance (ANOVA). Corresponding *p*-values of < 0.05 were considered significant.

## 3. Results and Discussion

### 3.1. Analysis of Collagen at Different Stages of Cross-Linking

**Figure 2:**
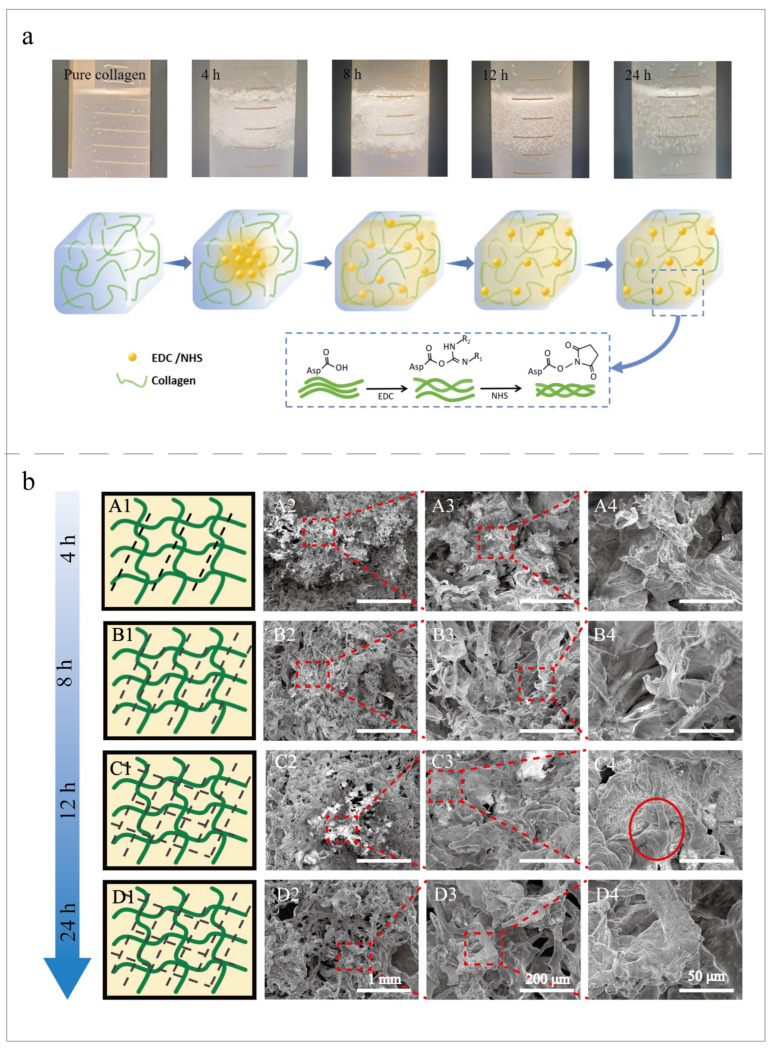
The morphology of the pure collagen and collagen at 4 h, 8 h, 12 h, and 24 h cross-linking times. (**a**) The macroscopic morphology of the cross-linked collagen and a schematic diagram of the diffusion process of the cross-linker. The block diagram is a schematic diagram of the principles of the EDC-/NHS-cross-linked collagen. (**b**) SEM images of the cross-linked collagen at the 1 mm (×100), 200 μm (×500), and 50 μm (×1500) scales. The red dashed boxes represent areas of the image that have increased magnification. (**A1**–**D1**) show collagen cross-linking diagrams. The green curve indicates the collagen fibers, and the black dashed line indicates the degree of cross-linking. The degree of cross-linking increased with increases in time. (**A1**–**A4**) show the cross-linking collagen for 4 h. (**B1**–**B4**) show the cross-linking collagen for 8 h. (**C1**–**C4**) show the cross-linking collagen for 12 h. The fibers had a smooth surface (**C4**, circle). (**D1**–**D4**) shows the cross-linking collagen for 24 h.

Firstly, according to the cross-linking process in natural bone, different time points were set for the cross-linking study. With an increase in the cross-linking time, the morphology and properties of collagen also changed. This study explored the characteristics of collagen at 4 h, 8 h, 12 h, and 24 h of cross-linking. At the macro level, cross-linked collagen samples were observed (Figure 2a). It was found that pure collagen without cross-linking appeared in the solution. The middle part of the collagen cross-linked for 4 h presented a relatively compact form, maintaining a certain fluidity and softness, but the distribution was not uniform. The morphology and distribution of the samples after 8 h of cross-linking were relatively dense and uniform. Cross-linked collagen was evenly and tightly distributed for 12 h. The samples that were cross-linked for 24 h were similar to the samples cross-linked for 12 h. This showed that, in the early stage of the cross-linking process, the cross-linking agent was still relatively concentrated in the middle of the collagen protein without diffusion and that a solid network was not formed. In the middle stage of the cross-linking, the cross-linking agent gradually diffused, resulting in a greater degree of condensation. In the later stage of cross-linking, the distribution of cross-linkers became uniform, which formed a dense network structure between the collagen molecules. The EDC cross-linked the collagen by binding carboxylic or phosphate groups to the primary amines. NHS improved the stability and efficiency of the cross-linked reactions (Figure 2a, block) [16].

The changes in the pore structure and fiber arrangement of the collagen samples at different cross-linking times were observed by SEM. After 4 h of cross-linking (Figure 2b(A1–A4)), it could be observed that the fiber structure was relatively loose and the pores between the fibers were large. After magnification, it was observed that the fibers began to form cross-links but still maintained a certain degree of independence. After 8 h of cross-linking (Figure 2b(B1–B4)), the pores began to become relatively small and the structure between the fibers became tighter. After magnification, the structure of the fiber network became more obvious, and the connections between the fibers were filamentous. After 12 h of cross-linking (Figure 2b(C1–C4)), the pores were further reduced, and the fiber network structure was tight. The cross-linking between the fibers was enhanced, and the filamentous connections increased to form lamellar networks. It was observed by SEM images that the pore diameter of the cross-linked sample slightly increased at 24 h (Figure 2b(D1–D4)). After magnification, it was observed that, although some of the pores were large overall, the structure between the fibers was relatively tight, which might indicate a very high degree of cross-linking. In conclusion, with an extension of the cross-linking time, the microstructure of collagen gradually went from tight to loose.

**Figure 3:**
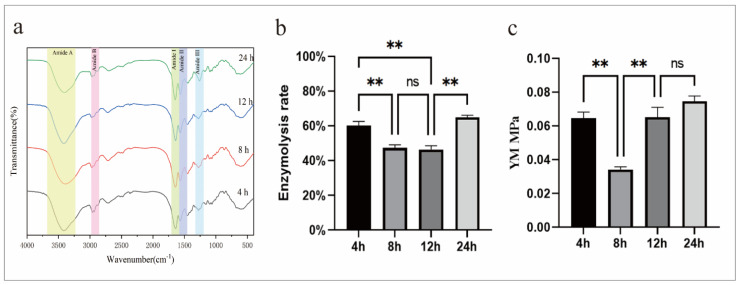
(**a**) The FTIR of collagen at 4 h, 8 h, 12 h, and 24 h cross-linking times. (**b**) The enzymolysis rate of collagen at 4 h, 8 h, 12 h, and 24 h cross-linking times. (**c**) The Young’s modulus of collagen at 4 h, 8 h, 12 h, and 24 h cross-linking times. ** *p* < 0.01, ns: no significant difference.

FTIR can determine the basic composition and molecular structure of a substance by detecting properties such as vibration and stretching at different frequencies. The absorption peaks of the classical structure of collagen are amide A, amide B, amide I, amide II, and amide III. The presence of these characteristic peaks proved the existence of post-cross-linked collagen structures (Figure 3a). The main vibration of the amide I band is the C=O stretching vibration and the N-H bending vibration. The main vibration of the amide II band is the C-N stretching vibration and the N-H bending vibration. According to the mechanism of the EDC-NHS cross-linking collagen, the amidation between the collagen molecules reduces the corresponding primary amino number and increases the amide bond. In the FTIR infrared spectra, the ratio of the absorption peak intensity between the amide II band and the amide I band decreases. It was found that, through calculation, with the increase in cross-linking time, the ratio of the absorption peak area between amide II band and amide I band decreased (Table 2). There was no difference in the ratio between 12 h and 24 h. This showed that the occurrence of cross-linking reaction increased with the increase in time within 12 h.

The enzymolysis rate of the collagen at different cross-linking times was obtained by an enzymolysis experiment (Figure 3b). It could be observed that the enzymolysis rate of cross-linking for 8 h and 12 h was significantly lower than that of cross-linking for 4 h. The results indicated that the time and degree of cross-linking increased and the ability to resist enzymatic hydrolysis increased. However, the enzymolysis rate of 24 h cross-linking increased. Combined with the highest YM of the samples in 24 h in the later mechanical tests, it was speculated that 24 h cross-linking might cause the structure to be too rigid and that it might even cause stress release during enzymatic hydrolysis. This caused some of the cross-linking bonds to break, thus increasing the enzymatic hydrolysis rate.

The Young’s modulus (YM) of collagen under different cross-linking times was obtained by a mechanical compression test (Figure 3c). It was observed that the YM decreased first and then increased. In the early stages of cross-linking, the formation of chemical bonds may lead to an increase in the tightness of the collagen structure. But the cross-linking agent had not been diffused and was concentrated in a small range, resulting in a high cross-linking degree and high elastic modulus in some regions. As the cross-linking time increased, the cross-linking agent diffused, and the distribution of the chemical bonds at this stage made the structure more prone to deformation, resulting in the elastic modulus being low. After 12 h of cross-linking, the cross-linking agent evenly diffused, the number of chemical bonds increased, the degree of cross-linking was high, and, therefore, the YM increased. There was no significant difference in the YM between 24 h of cross-linking and 12 h of cross-linking, indicating that the mechanical properties of collagen would become stable after 12 h of cross-linking.

### 3.2. Influence of the Dynamic Cross-Linking on the Crystal of Mineralized Collagen

In this study, the bionic mineralization of collagen in vitro under dynamic cross-linking was investigated according to the cross-linking and mineralization process of natural bone. Since 12 h is the time when HA begins to mature [17], collagen mineralization takes 12 h. Group A was cross-linked for 12 h and mineralized for 12 h. In Group B, the cross-linked mineralization was simultaneously carried out for 12 h. Group C was cross-linked for 4 h and then cross-linked and mineralized for 12 h. Group D was cross-linked for 8 h and then cross-linked for 12 h. During mineralization, the calcium ions presented in the solution combined with phosphate ions to form the mineral phase HA. Type I collagen then formed mineralized collagen fibril with minerals and arranged it into a fibrous array [18].

**Figure 4:**
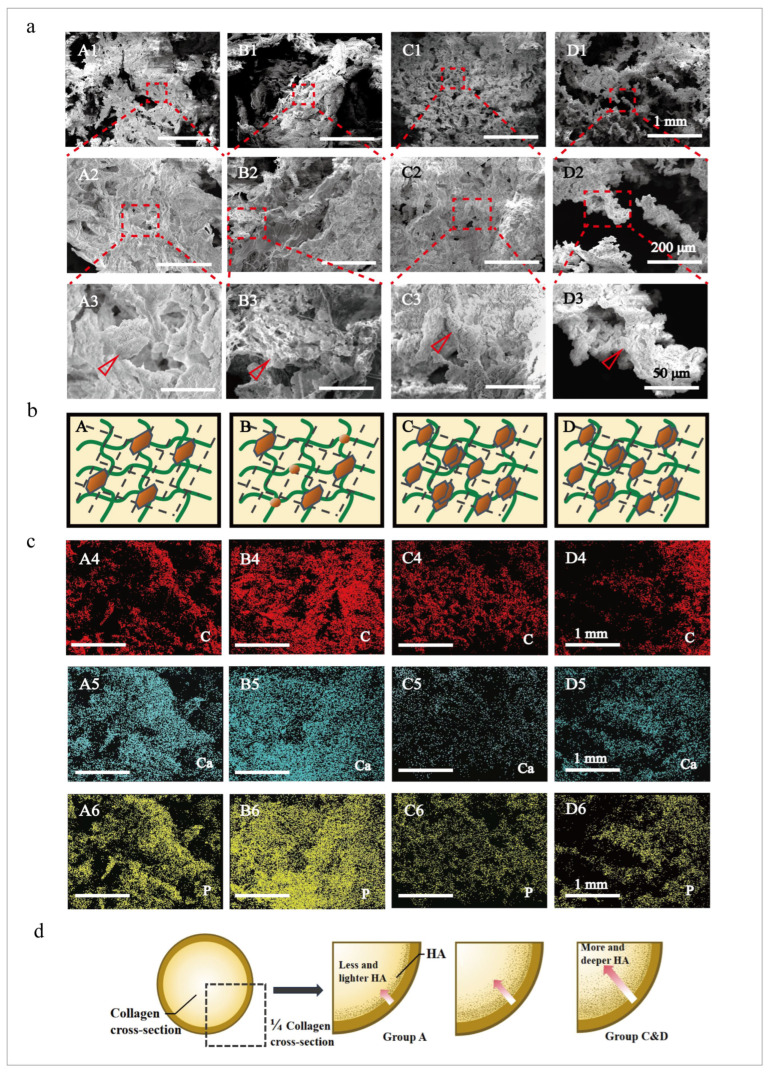
The morphologies of the mineralized collagen under different cross-linking conditions. (**a**) SEM images of the mineralized collagen at 1 mm, 200 μm, and 50 μm scales. A1–A3 are the images of Group A. B1–B3 are the images of Group B. C1–C3 are the images of Group C. D1–D3 are the images of Group D. Hydroxyapatite was found in all four groups. (A3–D3, triangles). (**b**) A schematic diagram of the mineralization process under different cross-linking conditions. Group A is mineralization after complete cross-linking. Group B is simultaneous cross-linked mineralization. Group C&D is simultaneous cross-linked mineralization after pre-cross-linking. (**c**) EDS images of mineralized collagen at 1 mm. C stands for carbon, Ca stands for calcium, and P stands for phosphorus. A4–A6 are the images of Group A. B4–B6 are the images of Group B. C4–C6 are the images of Group C. D4–D6 are the images of Group D. (**d**) A schematic diagram showing that the mineral mass of the dynamic cross-linking group was more deeply embedded in the mineralized collagen, while the mineral mass of the mineralized after cross-linking group was less and was more attached to the surface.

The morphology and mineral distribution of the mineralized collagen scaffolds under different cross-linking conditions were observed by SEM (Figure 4a). The pores of the mineralized collagen in Group A were loose, and the surface of the mineralized collagen was coated with minerals, forming flake hydroxyapatite. In Group B, the collagen fibers contained less mineral content and uneven distributions of mineralized particles. Flaky minerals were attached to the collagen surface, while small spherical minerals were attached to the collagen fibers. The mineralized collagen pores of Group C and Group D were relatively loose, which could better allow minerals to enter the collagen interior. A clear tangle of cross-linked collagen was observed. Minerals were heavily attached to collagen fibers. Therefore, it was speculated that cross-linking followed by mineralization causes the minerals to adhere to the collagen surface in large tracts. Simultaneous cross-linked mineralization resulted in an uneven distribution and low content of minerals. Dynamic-cross-linked mineralization after pre-cross-linking resulted in a uniform distribution and increased mineral content (Figure 4b).

The mineralized collagen elements were analyzed by EDS (Figure 4c). In Group A, Ca and P were widely distributed on the surface of the mineralized collagen, indicating that mineralization destroys the cross-linked structure, resulting in mineral adhesion on the collagen surface without penetrating into the collagen fiber. In Group B, Ca and P were widely and uniformly distributed on the surface of the mineralized collagen but did not enter the collagen interior. It was speculated that the cross-linking points were robbed when cross-linking and mineralization were carried out at the same time. The cross-linking effect was poor, and it could not provide better structural conditions for mineral deposition, so the mineralization effect was not good. The distribution of Ca and P in Groups C and D was consistent with the distribution of C, indicating that the simultaneous occurrence of mineralization and cross-linking after a longer period of time could make minerals grow and intertwine on the collagen. The minerals went deeper into the collagen fibers to form mineralized collagen with uniform mineralization. Therefore, it was speculated that the content of HA was less, and it was distributed on the collagen surface after cross-linking and mineralization. However, the dynamic cross-linking mineralization after pre-cross-linking resulted in high HA content, and it was also deep in the collagen fibers (Figure 4d).

**Figure 5:**
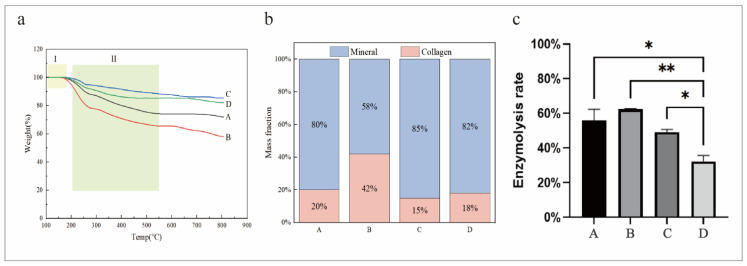
(**a**) The thermogravimetric curves of the mineralized collagen matrix. The yellow box I indicates a range of 100–180 °C, and the green box II indicates a range of 200–550 °C. (**b**) The mass fraction of each component of the composite mineralized collagen. (**c**) The enzymolysis rate of the mineralized collagen. * *p* < 0.05, ** *p* < 0.01.

The weight loss of the mineralized collagen under different cross-linking conditions was analyzed by TG, and the proportion of the inorganic and organic matter in the mineralized collagen was determined. As the temperature rose, the TG curve of the collagen could be used to distinguish the two weight loss steps. In the range of 100–180 °C, the bound water was first lost (Figure 5a, yellow frame I). In the range of 200–550 °C, the collagen chain was broken, and the structure was degraded (Figure 5a, green frame II). The thermal stability of Group B was poor, and the mineral content was low (Figure 5b). This indicated that simultaneous cross-linking and mineralization without pre-cross-linking affected the efficiency of the cross-linking and the quality of mineralization, resulting in a less stable structure. The thermal stability and mineral content of the pre-cross-linked (Groups C and D) samples were higher than those of Group A, which reflects that the dynamic cross-linking of pre-cross-linked materials contributes to the mineralization process, resulting in the formation of more stable and mineral-rich materials. In the process of pre-cross-linking followed by cross-linking and mineralization, cross-linking agents increase the connections between collagen fibers, which could provide more binding sites or stronger structural support, thus promoting mineral adsorption and deposition. In the process of dynamic cross-linking, the interactions between collagen fibers and cross-linking agents are still in progress, which might make the fiber structure maintain an “open state” to a certain extent. The opening of this structure might have increased the chance of contact between the minerals and collagen, allowing minerals to more easily permeate and deposit between collagen fibers, thus promoting mineralization efficiency. Optimizing the structural and functional properties of materials by regulating the synchronization of cross-linking and mineralization processes was critical, especially in applications that require simulations of natural mineralized environments such as bone regeneration.

Trypsin specifically hydrolyzes collagen by severing the lysine (Lys) and arginine (Arg) residues of the carboxylate terminal in collagen. The enzymolysis rate of mineralized collagen under different cross-linking conditions was obtained by enzymolysis test, and the percentage of the collagen and mineral content in the mineralized collagen was obtained (Figure 5c). The collagen structure of Group A was completely stabilized and densified before mineralization. The fully cross-linked collagen network was difficult to fully penetrate by minerals in the mineralization stage, resulting in uneven mineral distribution and a weak collagen network. The enzymolysis rate of Group B was high, indicating that a simultaneous cross-linking mineralization without pre-cross-linking would affect the effects of cross-linking and mineralization, thus reducing the stability of mineralized collagen. In contrast, the enzymolysis rate of Group D was significantly lower than that of Group A and B. These results indicate that pre-cross-linking made the collagen network stable and dense and that it provided enough space for mineral penetration. This initially stabilized collagen network cross-linked and mineralized with minerals at the same time, forming a more dense and stable structure, which enhanced the enzymatic resistance of the mineralized collagen. In addition, although the mineral content obtained was not completely the same, due to the different detection principles, the mineral content of Groups C and D was higher than that of Groups A and B, which was consistent with the results of the TG analysis, indicating that dynamic cross-linking could promote collagen mineralization.

### 3.3. Effect of Dynamic Cross-Linking on the Inorganic Substance Content of Mineralized Collagen

**Figure 6:**
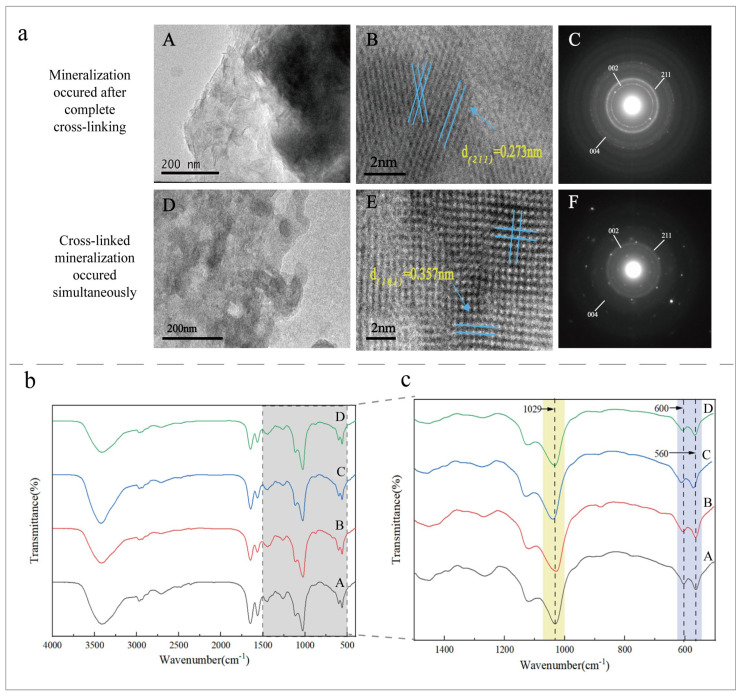
(**a**) TEM and SAED images of the cross-linked and then mineralized collagen and dynamic cross-linking of the mineralized collagen. (**A**–**C**) are the group of “Mineralization occurs after complete crosslinking”. (**D**–**F**) are the group of “Cross-linked mineralization occurs simultaneously”. (**b**) The FTIR of the mineralized collagen in each group. (**c**) The amplification of the gray area of (**b**) (500 cm^−1^–1500 cm^−1^).

The microstructure of the mineralized collagen under different cross-linking conditions was observed by TEM (Figure 6a). Evident HA lattice fringes could be observed. The SAED diffraction rings of mineralized collagens with complete cross-linking and collagens with simultaneous cross-linking mineralization contained specific diffusion rings corresponding to the (002), (211), and (004) reflections. Figure 6a(C,F) show that both of them had the characteristics of highly arranged HA nanocrystals.

FTIR can be used to analyze the composition of mineralized collagen, thereby determining functional groups based on the absorption peaks in the spectrum (Figure 6b). The O-P-O asymmetric absorption peaks near 1029 cm^−1^ and at 600 cm^−1^ and 560 cm^−1^ were vibrational absorption peaks of the phosphate groups, and they were presumed to form the HA material. The peak height and peak area of each group were calculated (Table 3). The positions of the HA peaks were basically the same, indicating that HA was well formed, but there were some differences in the peak intensity. There was no significant difference between the characteristic peaks of the A, B, C, and D groups at 560 cm^−1^ and 600 cm^−1^, while the characteristic peaks of Group D were the highest and the peak area was the largest at 1029 cm^−1^, indicating that the phosphate group concentration was higher and that the mineralization effect was better. According to the relative heights of the 560 cm^−1^ and 600 cm^−1^ peaks in the FTIR test and the valley between them (Figure 5c), the crystallinity of the mineralized collagen was semi-quantitatively calculated [19]. Moreover, the highest crystallinity of Group D indicated that more HA crystals with a certain regular arrangement had been formed. It showed that the mineralization degree of the cross-linked side had increased.

### 3.4. Effect of the Dynamic Cross-Linking on the Properties of Mineralized Collagen

**Figure 7:**
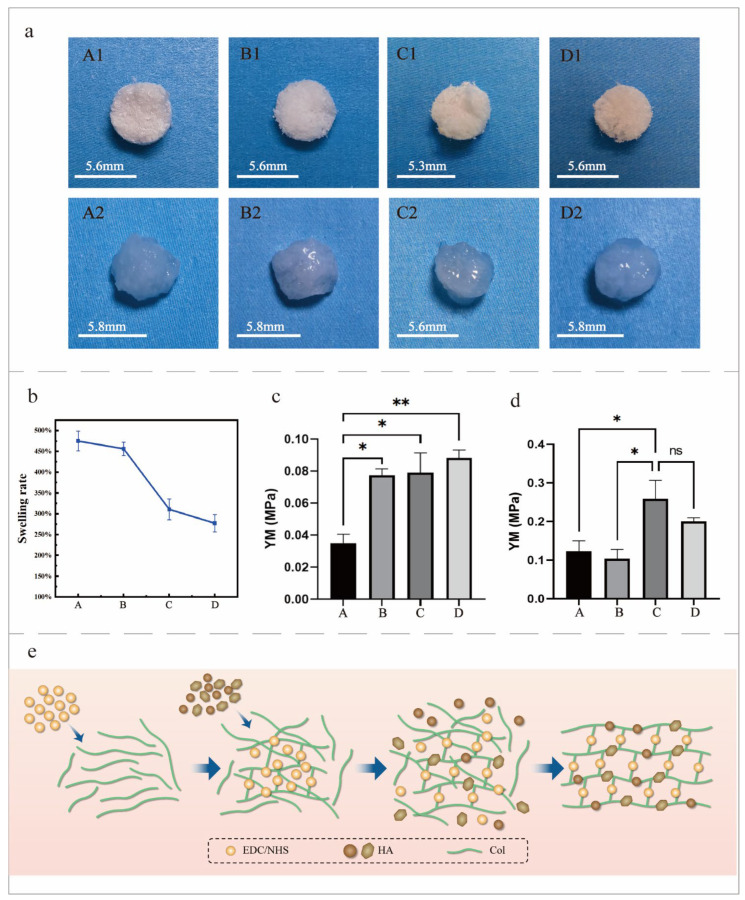
(**a**) Comparison of the mineralized collagen before and after swelling under different cross-linking conditions. (**A1**–**D1**) are the images before swelling, and (**A2**–**D2**) are the images after swelling. (**A1**–**A2**) are the images of Group A. (**B1**–**B2**) are the images of Group B. (**C1**–**C2**) are the images of Group C. (**D1**–**D2**) are the images of Group D. (**b**) The swelling rate of the mineralized collagen. (**c**) The Young’s modulus of the mineralized collagen. (**d**) The Young’s modulus of the mineralized collagen after swelling. (**e**) A schematic diagram of the dynamic-cross-linked mineralization after pre-cross-linking. * *p* < 0.05, ** *p* < 0.01, ns: no significant difference.

Swelling rate is an important parameter to characterize the structure of hydrogel network, and it depends on the degree of cross-linking [20]. The morphology of the mineralized collagen before and after swelling was observed under different cross-linking conditions (Figure 7a), and it was found that the volume of Groups A, B, C, and D increased little after swelling. Combined with the mechanical properties after swelling, it was indicated that the mineralized collagen could provide better mechanical properties and biocompatibility while maintaining a small volume change. This is essential for maintaining the stability of the organizational structure. The swelling rate of mineralized collagen under different cross-linking conditions was calculated (Figure 7b). It can be seen that the swelling rate was above 100%, indicating that the mineralized collagen had good swelling performance. The swelling rate of Groups C and D was significantly lower than that of Groups A and B, which was due to the increase in the cross-linking degree of the pre-cross-linked, dynamic, and cross-linked mineralized collagen. In addition, the cross-linking network became more dense, and the number of free hydrophilic groups that could interact with water decreased [21].

The elastic modulus of the mineralized collagen under different cross-linking conditions was measured by a mechanical compression test to evaluate the mechanical properties of the scaffold (Figure 7c). The elastic modulus of the mineralized collagen in Group A was significantly lower than that in the dynamic cross-linking group (B, C, and D) because the collagen structure after cross-linking was completely stable before the mineralization stage. This stability prevented mineral ions from entering the collagen matrix, resulting in poor mineralization and, therefore, the lowest Young’s modulus. Dynamic cross-linking could make mineral ions penetrate better and provide higher mineralization efficiency. Compared with Groups B and C, the Young’s modulus of Group D was the highest, indicating that cross-linking in a period of time provides a certain degree of stability for collagen mineralization and makes the collagen structure more easily permeated by mineral ions in the subsequent simultaneous cross-linking and mineralization process. Niu et al. [22] prepared cyclic, compressive-stress mineralized collagen, whose YM is able to reach 2 MPa. In contrast, the YM of the dynamic-cross-linked mineralized collagen prepared in this study was not high enough, and the mechanical properties need to be improved. Compared with the mineralized collagen before swelling, the elastic modulus of the mineralized collagen after swelling increased, indicating that the mineralized collagen evolved in a more compact and orderly direction (Figure 7d).

The dynamic-cross-linked collagen mineralization mechanism after pre-cross-linking is shown in the Figure 7e. The collagen cross-linked the frame first, and then the collagen cross-linked the frame while stacking minerals. This allowed the collagen minerals to cross-link deep into the collagen. Therefore, the mineralization effect was better (Figure 7e).

The innovation of this study was that it combined the nonlinear characteristics of natural bone growth to explore the effect of the cross-linking processes on mineralization in stages. In our study, dynamic cross-linking refers to a cross-linking process that simultaneously occurs with the mineralization process. The mineralized collagen of the EDC-NHS cross-linking prepared by Jeremy Elias et al. [23] had a mineral content of 52%, while the mineralized collagen of the dynamic cross-linking prepared by us had a mineral content of 85%. Through comparison, it can be seen that dynamic cross-linking can increase the mineral content of mineralized collagen. Having said this, we did not have enough control over the dynamic process. In another study, dynamic cross-linked hydrogels were constructed into polymer cross-linked network gels with excellent self-healing properties using dynamic covalent bonds [24]. The cross-linking process used in that study was more controlled. In further studies, we plan to combine dynamic, covalent-bond cross-linking with the cross-linking process to prepare a more controllable dynamic cross-linked mineralized collagen.

## 4. Conclusions

In natural bone, the process, i.e., the dynamic cross-linking process, of collagen fibril formation and collagen fiber mineralization is continuous. Based on this, this study prepared and explored mineralized collagen under the different cross-linking and mineralizing processes of natural bone. Compared with the traditional mineralized collagen, with 12 h of cross-linking followed by 12 h of mineralization, and the fully synchronized cross-linked collagen, the dynamic cross-linked mineralized collagen with 8 h of pre-cross-linking saw improved mineral content, enzyme stability, and mechanical properties, as well as a reduced swelling rate. It is suggested that dynamic cross-linking by pre-cross-linking enables minerals to more easily permeate and deposit between collagen fibers, thus promoting mineralization efficiency and improving the mechanical strength of mineralized collagen. In this study, the dynamic cross-linking regulation of biomimetic collagen mineralization was realized in vitro, and it is expected to promote in-depth explorations of the biomimetic mechanism and provide new ideas for designing mineralized collagen scaffolders with better bone repair ability. In the future, the plan is to combine dynamic, covalent-bond cross-linking with cross-linking technology to prepare more controllable dynamic-cross-linked mineralized collagen, and we also intend to explore the biocompatibility of the samples.

## Figures and Tables

**Table 1:** The cross-linking and mineralization time of each experimental group.

Groups	Pre-Cross-Linking	Mineralization	Dynamic Cross-Linking
A	12 h	12 h	0 h
B	0 h	12 h	12 h
C	4 h	12 h	8 h
D	8 h	12 h	4 h

**Table 2:** The peak area and ratio of amide I and amide II at 4 h, 8 h, 12 h, and 24 h cross-linking times.

Groups	Amide I Peak Area	Amide II Peak Area	The Peak Area Ratio
4 h	53.571	28.904	0.540
8 h	55.217	26.369	0.478
12 h	56.287	25.040	0.445
24 h	56.837	25.336	0.446

**Table 3:** The height and area of the 1029 cm^−1^ peak and degree of crystallinity.

Groups	1029 cm^−1^ Peak Height	1029 cm^−1^ Peak Area	Degree of Crystallinity
A	0.546	27.899	3.53
B	0.558	31.357	3.99
C	0.562	31.157	3.90
D	0.605	32.809	4.56

## Data Availability

The original contributions presented in the study are included in the article, further inquiries can be directed to the corresponding author.
